# Binding kinetics of cariprazine and aripiprazole at the dopamine D_3_ receptor

**DOI:** 10.1038/s41598-018-30794-y

**Published:** 2018-08-21

**Authors:** Annika Frank, Dóra J. Kiss, György M. Keserű, Holger Stark

**Affiliations:** 1 Institute of Pharmaceutical and Medicinal Chemistry, Heinrich Heine University Düsseldorf, Duesseldorf, Germany; 2 Medicinal Chemistry Research Group, Research Centre for Natural Sciences, Hungarian Academy of Sciences, Budapest, Hungary; 3 ELTE Eötvös Loránd University, Doctoral School of Chemistry, Budapest, Hungary

## Abstract

The dissociation behaviours of aripiprazole and cariprazine at the human D_2_ and D_3_ receptor are evaluated. A potential correlation between kinetics and *in vivo* profiles, especially cariprazine’s action on negative symptoms in schizophrenia, is investigated. The binding kinetics of four ligands were indirectly evaluated. After the receptor preparations were pre-incubated with the unlabelled ligands, the dissociation was initiated with an excess of [^3^H]spiperone. Slow dissociation kinetics characterizes aripiprazole and cariprazine at the D_2_ receptor. At the D_3_ receptor, aripiprazole exhibits a slow monophasic dissociation, while cariprazine displays a rapid biphasic behaviour. Functional ß-arrestin assays and molecular dynamics simulations at the D_3_ receptor confirm a biphasic binding behaviour of cariprazine. This may influence its *in vivo* action, as the partial agonist could react rapidly to variations in the dopamine levels of schizophrenic patients and the ligand will not quantitatively dissociate from the receptor in one single step. With these findings novel agents may be developed that display rapid, biphasic dissociation from the D_3_R to further investigate this effect on *in vivo* profiles.

## Introduction

Schizophrenia is a group of neurological diseases characterized by specific symptom complexes. Positive symptoms, which are exaggerated in schizophrenic patients, include hallucinations or delusions. Negative symptoms (*e*.*g*. depression or anhedonia) represent the impairment of healthy cognitive behaviour. The origin of the symptoms is not clarified, but a neurotransmitter dysregulation in different parts of the brain is likely. Hyperactive dopamine transmission and increased D_2_ receptor (D_2_R) activation may cause positive symptoms. Hypoactive dopamine transmission and hence less D_1_ receptor activation in the prefrontal areas may be responsible for negative symptoms^1^. D_3_ receptors (D_3_Rs) seem to contribute to the origin of negative symptoms^2^. To date schizophrenia is treated only symptomatically. Typical first generation antipsychotic drugs reduce the positive symptoms but display severe and therapy limiting adverse drug effects (ADE) like extrapyramidal motoric symptoms (EPMS). The second generation, also known as atypical antipsychotic drugs reduce these adverse effects, while remain highly effective against positive symptoms. Aripiprazole, an atypical antipsychotic agent, exhibits good efficacy on the positive symptomatic of schizophrenia and a rather beneficial ADE profile. However it is not able to treat the negative symptoms of the disease sufficiently. Cariprazine, a novel antipsychotic agent^3,4^, entered the US market in 2016 and obtained European Medicines Agency (EMA) approval in May 2017. Unlike most other antipsychotics it is effective against negative symptoms in clinical trials^5^, although the mechanism of this action remains elusive.

Distinct residence times (RTs) are discussed to influence drug profiles *in vivo*^6^, although proof of this hypothesis remains pending. As the RT (1/k_off_) determines the duration of receptor occupancy it may influence signalling pathways or ADE^7,8^. Several studies address the RTs of antipsychotic drugs at the D_2_R and the correlation to efficacy or ADE^9^, however, to date such approaches have not been made for the D_3_R. A recent review highlighted the relevance of conformational receptor states and biased signalling at G-protein coupled receptors (GPCRs)^10^. With increasing insights into the crystal structure and signal transduction of GPCRs, it becomes more important to evaluate pharmacological characteristics of novel agents. We hypothesize that cariprazine’s dissociation behaviour elucidates possible mechanisms for its action on negative symptoms in schizophrenia. Its profile is compared to the partial agonist aripiprazole, the antagonist spiperone and the agonist rotigotine. In doing so, the understanding of ligand binding to the D_3_R could be improved and drug development might benefit from clinically optimized drugs, focused on specific symptoms.

## Results

### [^3^H]spiperone kinetic experiments

Dissociation rate constants (k_off_) of [^3^H]spiperone are 0.013 min^−1^ (±0.003 min^−1^) at the D_2_R and 0.033 min^−1^ (±0.019 min^−1^) at the D_3_R. The k_off_ of unlabelled spiperone in the indirect assay matches the k_off_ of [^3^H]spiperone at both receptors (Tables 1 and 2), supporting the applicability of the dilution method.

**Table 1:** D_2_R k_off_ and equilibrium dissociation constants (±s.d.) of the unlabelled ligands.

Drug	k_off_ [min^−1^] ± s.d. {n}	pK_i_ ± s.d. (K_i_ [nM]) {n}
Aripiprazole	0.026 ± 0.001 {3}	8.95 ± 0.06 (1.1) {3}
Cariprazine	0.041 ± 0.002 {3}	8.80 ± 0.12 (1.6) {3}
Rotigotine	0.113 ± 0.029 {3}	7.73 ± 0.10 (18.5) {4}
Spiperone	0.028 ± 0.018 {3}	10.01 ± 0.29 (0.1) {4}

pK_i_ are determined by radioligand displacement assays. D_2s_R membrane preparations were incubated with [^3^H]spiperone and the compound. k_off_ are determined by radioligand dilution assays. D_2s_R membrane preparations were pre-incubated with the compound. Afterwards dissociation was initiated with an excess of [^3^H]spiperone. Results are means ± s.d. at least performed with n = 3 independent experiments at room temperature.

**Table 2:** D_3_R k_off_ and equilibrium dissociation constants (±s.d.) of the unlabelled ligands.

Drug	Monophasic fit	Biphasic fit		pK_i_ ± s.d. (K_i_ [nM]) {n}
	k_off_ [min^−1^] ± s.d. {n}	k_off1_ [min^−1^] ± s.d. {n}	k_off2_ [min^−1^] ± s.d. {n}	
Aripiprazole	0.05 ± 0.02 {3}	0.03 ± 0.02 {3}	0.15 ± 0.08 {3}	8.13 ± 0.19 (7.3) {5}
Cariprazine	0.16 ± 0.04 {9}	0.03 ± 0.01 {8}	0.63 ± 0.38 {8}	9.24 ± 0.18 (0.6) {6}
Rotigotine	0.07 ± 0.02 {4}	0.04 ± 0.02 {3}	0.18 ± 0.01 {3}	8.44 ± 0.26 (3.7) {3}
Spiperone	0.05 ± 0.02 {3}	0.02 ± 0.01 {2}	0.47 ± 0.35 {2}	8.96 ± 0.28 (1.1) {4}

pK_i_ are determined by radioligand displacement assays. D_3_R membrane preparations were incubated with [^3^H]spiperone and the compound. k_off_ are determined by radioligand dilution assays. D_3_R membrane preparations were pre-incubated with the compound. Afterwards dissociation was initiated with an excess of [^3^H]spiperone. Results are means ± s.d. at least performed with n = 3 independent experiments at room temperature. k_off_ displays the k_off_ derived by the monophasic fit, while k_off1_ and k_off2_ are the two k_off_ derived by the biphasic fit.

### D_2_R kinetic experiments

**Figure 1:**
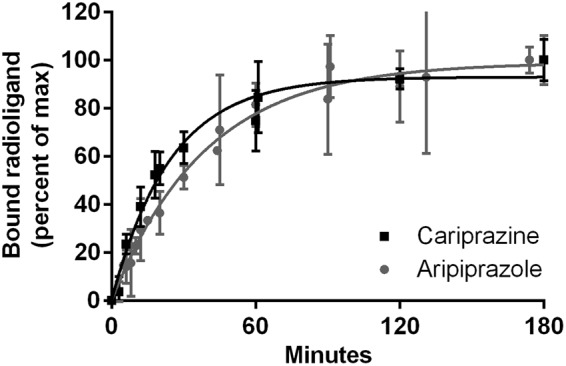
Dissociation of aripiprazole and cariprazine from the D_2_R, determined by dilution assays. After pre-incubating D_2s_R membrane preparations with the compounds, dissociation was initiated with an excess of [^3^H]spiperone. Figure displays normalized values globally fitted for all experiments (n = 3, triplicates) given as mean with s.d.

**Figure 2:**
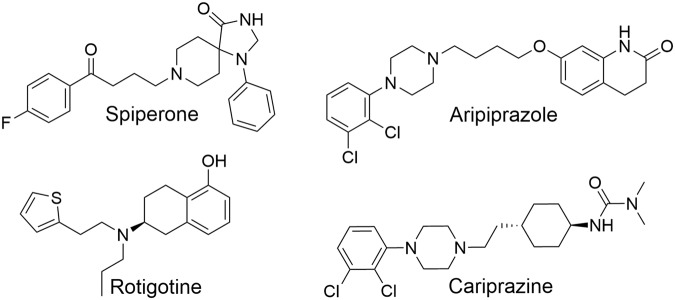
Chemical structures of the evaluated ligands.

Aripiprazole and cariprazine share similar RTs at the D_2_R with 38 min for aripiprazole and slightly faster (24 min) for cariprazine (Table 1, Fig. 1). These results are in accordance to those of Klein Herenbrink, who found only slight differences at the D_2_R^7^. The k_off_ of aripiprazole is concordant with studies performed on rat^8,11^ and human receptors^12,13^ and the slow dissociation of spiperone^7,14–16^ is also corroborated. References, predicting a fast k_off_ of aripiprazole from the D_2_R^7,17^ and a slow k_off_ for rotigotine^18^, are not confirmed. The measured RTs at the D_2_R correlate with the drugs’ affinities (radioligand displacement assays, pK_i_ Table 1). Spiperone and aripiprazole (highest affinities) display the longest RTs, while rotigotine (lowest affinity) shows the fastest dissociation from the D_2_R. This correlation of binding kinetics and affinities at the D_2_R was demonstrated by Kapur and Seeman^14^. Spiperone, aripiprazole and cariprazine (slow k_off_) share a large, lipophilic aromatic motif, connected through a linker to a second motif with different hydrogen-bond acceptor and donor groups (Fig. 2), while rotigotine is missing this diverse motif. In the presented study the antagonist and the partial agonists display a slow dissociation, while the full agonist rotigotine shows fast dissociation from the D_2_R, which is supported by their structural and binding properties.

### D_3_R kinetic experiments

**Figure 3:**
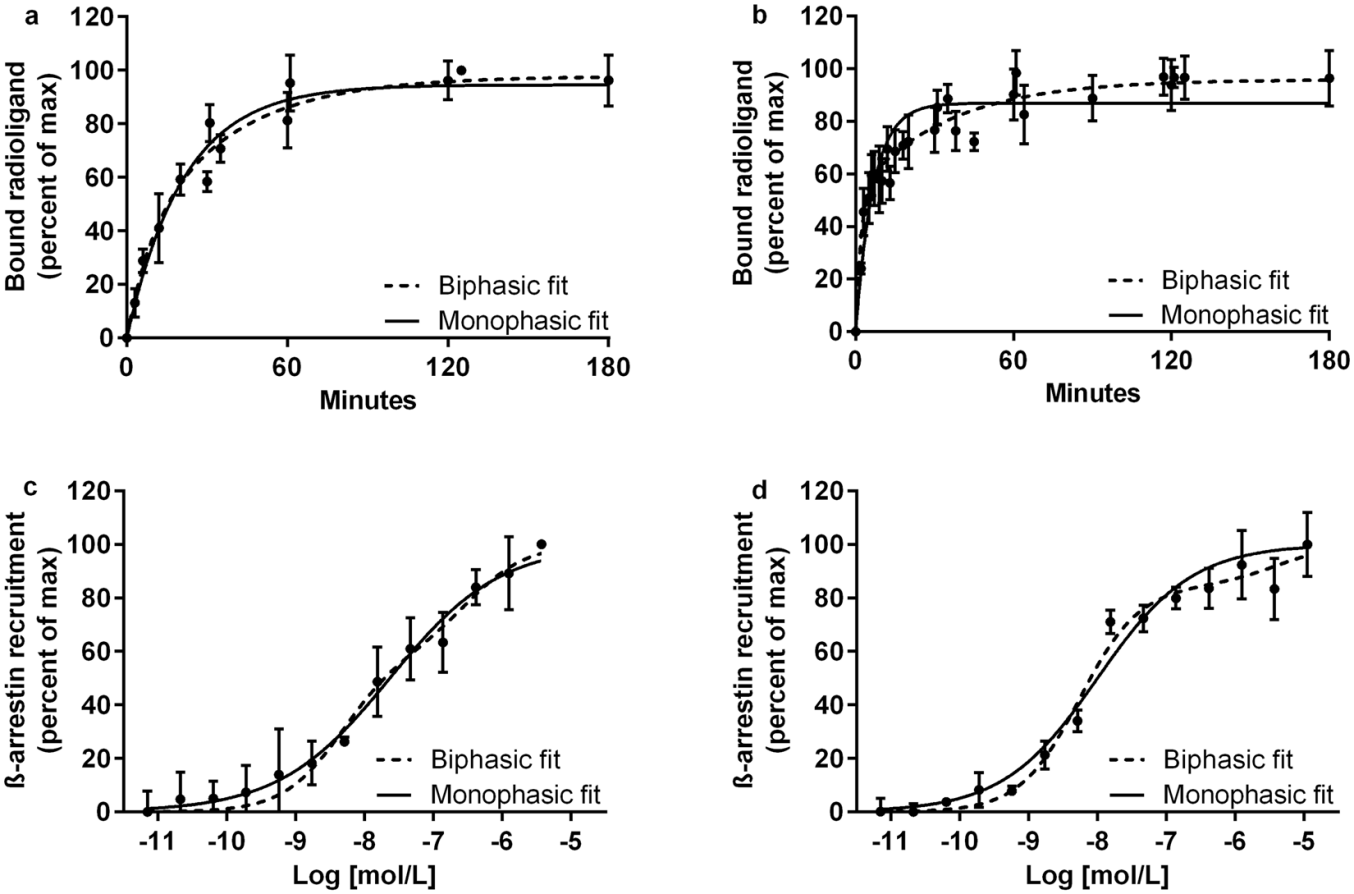
Dissociation profiles and ß-arrestin recruitment of aripiprazole and cariprazine at the D_3_R. (**a**,**b**) Dissociation of aripiprazole (**a**) and cariprazine (**b**) from the D_3_R, determined by dilution assays. D_3_R membrane preparations were pre-incubated with the compounds. Dissociation was initiated with an excess of [^3^H]spiperone. Figure displays normalized values globally fitted for all experiments (n = 3/9, triplicates) given as mean with s.d. (**c**,**d**) ß-arrestin activation of aripiprazole (**c**) and cariprazine (**d**) at the D_3_R. U2OS cells were incubated with partial agonists for 90 min. Data is normalized, globally fitted for all experiments (n = 2, duplicates) given as mean with s.d.

The evaluation of k_off_ at the D_3_R reveals different dissociation behaviours of aripiprazole and cariprazine (Table 2, Fig. 3a,b). Cariprazine displays a rapid biphasic dissociation behaviour (p < 0.0001) that is not sufficiently described by a monophasic fit. Dissociation of aripiprazole is slower and better described by a monophasic fit (p = 0.0925). Evaluation of the antagonist spiperone also reveals a slow, but biphasic (p < 0.0001) dissociation, while the agonist rotigotine displays a monophasic (p = 0.0688) dissociation, not significantly faster than aripiprazole or spiperone. The biphasic dissociation of cariprazine is not altered by the addition of guanosine 5′-[β,γ-imido]triphosphate (Gpp(NH)p), the absence of sodium in the buffer or the use of another radioligand ([^3^H]raclopride).

### Functional binding assays at the D_3_R

ß-arrestin 2 recruitment assays result in EC_50_ values of 23.7 nM for aripiprazole and 10.2 nM for cariprazine (monophasic fit). However, a biphasic fit of cariprazine is more appropriate (p = 0.0004) with EC_50_ values of 5.52 nM and 4.19 µM (Fig. 3c,d). Both are partial agonist in the tested system with ca. 30% of E_max_. A statistically non-significant biphasic binding is observed for cariprazine in radioligand displacement studies with pK_i_ of 8.71 and 11.91. In case of aripiprazole, the biphasic fit is not applicable.

### Molecular Modelling

**Figure 4:**
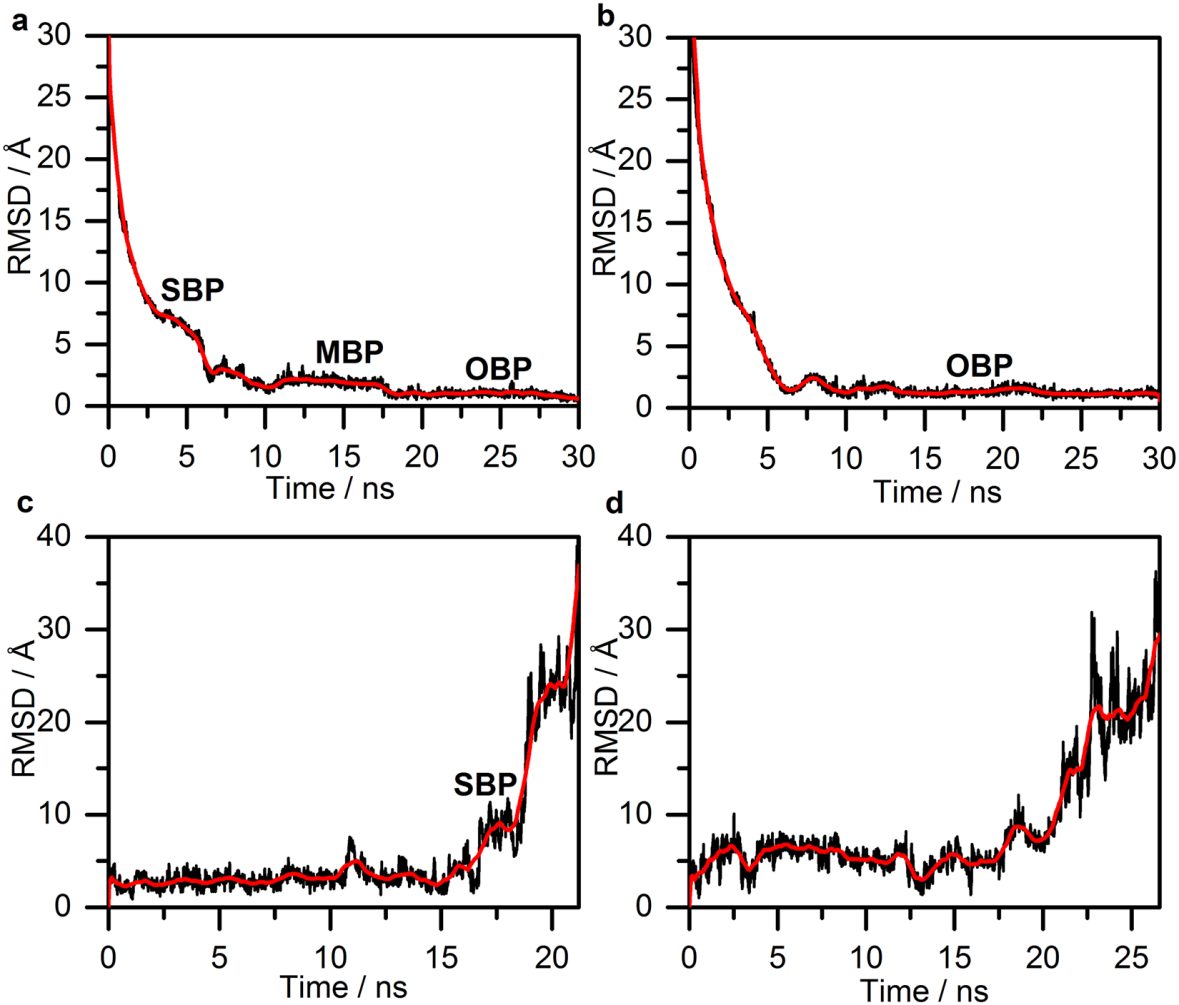
Ligand heavy atom RMSD during the binding (**a**: cariprazine, **b**: aripiprazole) and unbinding (**c**: cariprazine, **d**: aripiprazole) simulations. Black line corresponds to the calculated values, the smoothed curve is calculated as moving average for 100 points for better visualization. The separate phases of the binding are indicated on the graph.

**Figure 5:**
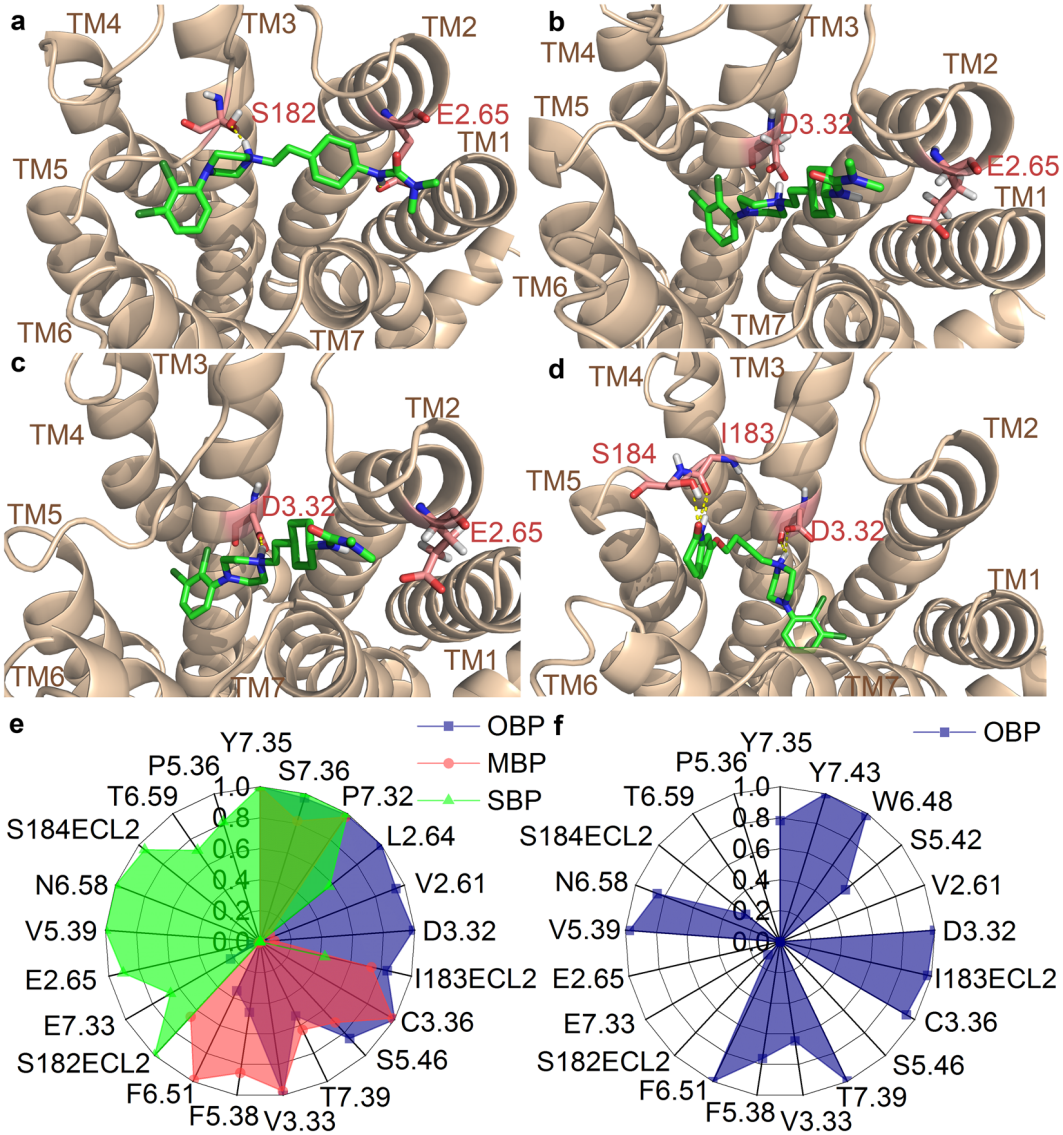
Binding poses and frequent interactions during binding simulations. (**a**–**d**) Cariprazine in the secondary binding pose (**a**, SBP), metastable binding pose (**b**, MBP) and the final binding pose (**c**, OBP) and aripiprazole’s final binding pose (**d**, OBP). The complexes are visualized from the top, the protein is represented as ribbon, the ligands as green sticks and important residues as light red sticks. Hydrogen bonds are shown as dashed yellow lines. (**e**,**f**) Most frequent interactions of cariprazine in the SBP (**e**, green), MBP (**e**, red) and OBP (**e**, blue) and aripiprazole in the OBP (**f**, blue).

**Figure 6:**
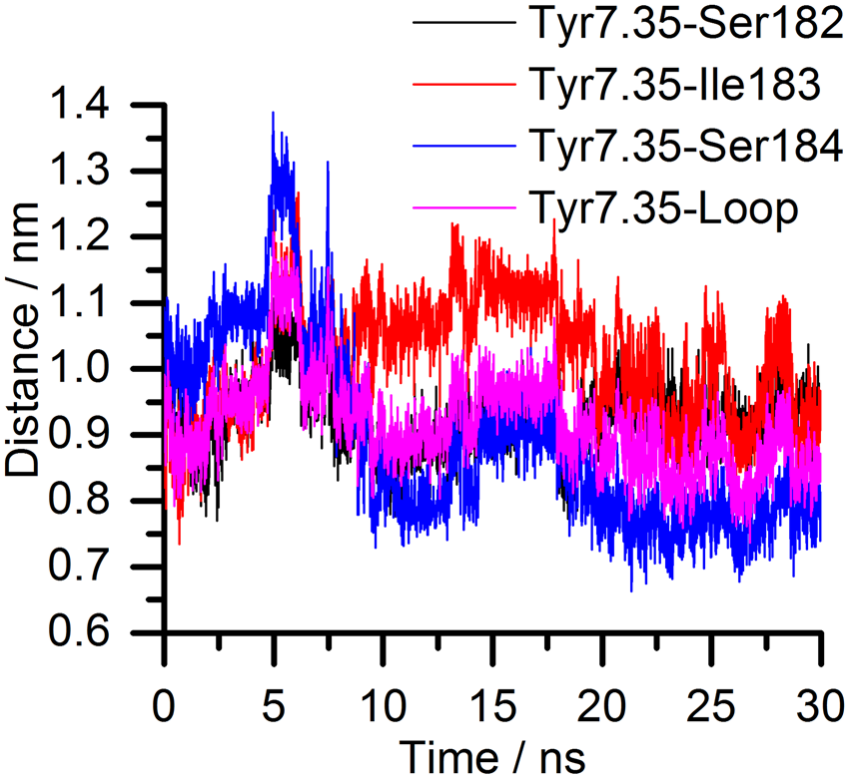
Tyr7.35 and ECL2 lock movement during the binding of cariprazine. Distances between the centre of masses of Tyr7.35 and ECL2 residues Ser182, Ile183, Ser184 and the centre of mass of the three residues together representing the loop position in the cariprazine binding trajectory to the D_3_R.

The root-mean-square deviation of atomic positions (RMSD) against the timeframe in binding trajectories are consistent with the observed biphasic kinetics of cariprazine (Fig. 4a) and the one-phase kinetics of aripiprazole (Fig. 4b). Upon reaching the receptor surface cariprazine contacts extracellular loop (ECL) 2 and ECL1, which are guiding it towards the entrance of the binding site. The 3–5 ns period (Fig. 4a) corresponds to the secondary binding pose (SBP), where cariprazine interacts with extracellular surface residues, mainly on ECL2, the top part of the transmembrane region (TM) 2 and TM5-7 (Fig. 5a). Key hydrogen-bonds (H-bonds) with Ser182^ECL2^ and Glu2.65 and hydrophobic interactions (*e*.*g*. Val5.39, Pro5.36) contribute to the stabilization (Fig. 5e). Tyr7.35 helps to isolate the ligand from the orthosteric binding site. After 5 ns cariprazine rearranges in the receptor cavity, enters the orthosteric site, transiently stabilized in a metastable binding pose (MBP, 10–17.5 ns, Fig. 4a) then reaches the final, orthosteric binding pose (OBP, 20–30 ns, Fig. 4a). These two positions resemble regarding the orientation of the ligand (Fig. 5b,c), however the occupancy of the key H-bond interaction with Asp3.32 increases from 10% (MBP) to 99% (OBP). Hydrophobic interactions (e.g. Val3.33, Ile183^ECL2^, Phe6.51, Phe5.38, Leu2.64 and Tyr7.35) also contribute to the stabilization (Fig. 5e) of the binding mode. Similar to previous molecular dynamic (MD) studies of D_3_R ligands^19^, Tyr7.35 and the ECL2 residues Ser182, Ile183, Ser184 function as a lock, facilitating the entrance of the ligand into the binding pocket (Fig. 6). The recently published high resolution structure of the D_2_R confirms the impact of Ile184 (Ile183 in the D_3_R) on the binding kinetics of bivalent ligands (ligands bearing two pharmacophores connected by a linker^13,20–22^). Aripiprazole also first interacts with the ECL2 of the receptor, then forms a π–π stacking interaction with Tyr7.35. The lock opens and the ligand enters the binding site. A stable intermediate state was not observed (Fig. 4b); the ligand proceeds till it reaches the OBP and forms the H-bond interaction with Asp3.32, which remains intact (10–30 ns). The orientation of the ligand (Fig. 5d) varies compared to cariprazine, but similar interactions were also detected (Fig. 5f). Both H-bonds (Asp3.32, Ile183^ECL2^) and hydrophobic interactions (e.g. Trp6.48, Phe6.51, Tyr7.43) stabilize its binding mode. Stability assessments and unbinding simulations suggest that aripiprazole might also reorient in the binding pocket to a similar pose as cariprazine.

Even though the unbinding simulations offer less reliable structural insight, the trajectories display some characteristic dissociation features. Cariprazine relocates into the SBP after 17 ns (Fig. 4c) and fully dissociates after 21 ns. During the unbinding of aripiprazole (Fig. 4d) we could not observe well defined stages, the higher fluctuations in the RMSD values show less stabilization and the shape of the graph resembles more a one-phase dissociation kinetics.

## Discussion

Cariprazine and aripiprazole display similar dissociation behaviours at the D_2_R, but differ at the D_3_R. Aripiprazole exhibits similar RT at the D_3_R and the D_2_R, whereas cariprazine shows the shortest RT and the most pronounced biphasic behaviour of the tested ligands at the D_3_R. This could influence cariprazine’s *in vivo* action, as it can react rapidly to variations in the dopamine level. Recent research revealed that metastable receptor states may determine binding orientation of bivalent drugs in MD simulations^22^ and may influence their binding kinetics^23^. Our findings suggest that cariprazine’s biphasic properties are connected to a metastable binding pose. Different binding poses may influence a biased agonism at the D_3_R, by stimulating different signalling pathways depending on the natural ligand’s concentration. It has to be mentioned that there may be other explanations for the biphasic *in vitro* behaviour. Given the high affinity and the fast k_off_ from the D_3_ receptor, the k_obs_ should be fast, which could result in rebinding effects in the assay. To check this possibility, different concentrations of cariprazine (0.2 and 40 times K_i_ value, n = 1) were evaluated, however, the biphasic nature of its dissociation remained. Although other models may also describe the obtained data, the combination of the experimental data and the MD simulations strengthen a biphasic *in vitro* dissociation profile of cariprazine and a pronounced interaction with different receptor sites. Various studies have shown that the ß-arrestin pathway at the D_2_R plays an important role in clinical efficacy of antipsychotics. Genetic ß-arrestin depletion led to a loss in antipsychotic activity of tested agents with an increase in motoric ADE^24^. A BRET assay revealed the antagonism of antipsychotic drugs, including aripiprazole, on the D_2_R mediated ß-arrestin recruitment^25^. Given the biphasic nature of cariprazines ß-arrestin recruitment and dissociation from the D_3_R, increases in local dopamine concentration may not lead to a complete displacement of the ligand, but a certain amount will be able to act at the target for a longer period.

A recent review^26^ criticizes the use of the RT as sole explanation of a drug’s profile and claims for individual evaluation of its impact. In the case of antipsychotic agents however, the correlation between RT and drug profiles seems to be a reasonable hypothesis for different *in vivo* profiles^9^. Cariprazine displays a different dissociation profile than other tested ligands and shows concurring properties in functional assays and MD simulations at the D_3_R. Its profile differs especially from aripiprazole, also a highly active antipsychotic, only lacking the effectivity on the negative symptoms. Therefore, the characteristic receptor interaction may be one possible cause behind the efficacy of cariprazine on the negative symptoms of schizophrenia.

Although the clinical effects of cariprazine seem to correlate to its binding behaviour at the D_3_R, there may be additional factors. The translation of an *in vitro/in silico* biphasic behaviour to *in vivo* effects remains to be elucidated and requires more research on the origin of negative symptoms. However, with these findings, it is possible to rationally develop compounds with biphasic kinetics and characterize them *in vivo* regarding their effects on the negative symptomatic. Within this work, we show that:

- Cariprazine and aripiprazole share similar dissociation properties at the D_2_R
- Cariprazine and aripiprazole display different dissociation profiles at the D_3_R
- Cariprazine dissociates faster from the D_3_R than from the D_2_R
- Cariprazine exhibits biphasic kinetics at the D_3_R

- The biphasic behaviour is also observed in functional assays with cariprazine
- MD simulations support the experiments with structural interpretation.

Recent work^13^ emphasizes the significance of evaluating and comparing binding modes to understand and advance drug development. By elucidating the binding profile of cariprazine the insights on receptor interaction at the D_3_R were broadened and future evaluation of novel antipsychotic drugs with special focus on the negative symptoms has gained an interesting aspect. By this, a next step in the understanding and improvement of antipsychotic therapy was made and future approaches may benefit from specialized agents for this diverse symptom complex.

## Methods

### Cell culture and membrane preparation of CHO cells expressing the hD_2s_R and the hD_3_R

Cell culture and membrane preparations were performed as reported previously with modifications^27^. CHO cells stably expressing the human dopamine D_2short_R or D_3_R were cultured in DMEM (with 1% glutamine, 10% FBS, and 1% penicillin/streptomycin for D_2;_ 1% glutamine, 10% dialysed FBS for D_3_). CHO-D_2_ cells were collected in PBS buffer, CHO-D_3_ cells in medium and centrifuged at 3,000 × g for 10 min at 4 °C. The pellet was resuspended in binding buffer (1 mM MgCl_2_, 1 mM CaCl_2_, 5 mM KCl, 120 mM NaCl and 50 mM Tris, pH 7.7), disrupted and centrifuged at 23,000 × g for 30 min (4 °C). The resulting pellet was stored in binding buffer at −80 °C.

### Radioligand displacement assays at the hD_2_R and the hD_3_R

Displacement assays were performed as reported previously^28^ with modifications. Briefly, membrane preparations (D_2s_R: 25 μg/well; D_3_R: 20 µg/well) were co-incubated with [^3^H]spiperone (0.2 nM) and the test ligand. Nonspecific binding (NSB) was measured with haloperidol (10 µM) and separation of bound radioligand was performed using VE-water. Assays ran in triplicates at least in three independent experiments. Data was analysed using non-linear regression and equation “one site competition”. The K_i_ values were calculated from the IC_50_ values using the Cheng-Prusoff equation^29^.

### Determination of the [^3^H]spiperone dissociation rate constants at the D_2_R and the D_3_R

The k_off_ of [^3^H]spiperone (RL) was measured, using an excess of haloperidol^30^. Cell preparations (D_2s_R: 25 μg/well; D_3_R: 20 µg/well) were incubated (120 min, 250 rpm) with 0.2 nM RL in 0.2 mL. The dissociation was initiated at different time points with an excess of haloperidol (0.4 mM stock, 15 µL to prevent dilution). Assays ran in quadruplicates with eleven time points with n = 4 at the D_2_R and n = 7 at the D_3_R. NSB was determined with haloperidol (10 μM), total binding with 0.2 nM RL for the total time of the experiments. Bound RL was separated as described above. Binding data was analysed using non-linear regression and fitting to “one phase exponential decay”.

### Determination of unlabelled ligands dissociation rate constants at the D_2_R and the D_3_R

The k_off_ of unlabelled ligands (UL) were measured indirectly by the dilution method, similar as described previously^30,31^. 50 µL of the membrane preparations (D_2s_R: 25 μg/well; D_3_R: 20 µg/well) were incubated with the UL at four times its K_i_ value for 120 min. Afterwards the dissociation was initiated at different time points with an excess of RL (150 µL, 20 times its K_D_ value). This dilution results in the majority of the receptors being occupied by RL at the end of the experiment. As the RL may only bind when the UL has dissociated, k_obs_ of the RL reflects the k_off_ of the UL (Supplementary Fig. S1). To ensure, that the association of the RL occurs without delay to the dissociation of the UL, the concentration of the RL has to be very high (*e*.*g*. 20 times K_D_ value). This model assumes that RL and UL bind to the same binding site. Assays ran in triplicates with eight time points with at least n = 3 independent experiments. Total binding was measured in the absence of UL; at the end of the experiments 80–100% of the total binding were achieved (spiperone and rotigotine 70% at the D_3_R). Rotigotine was measured at 5 times its K_i_ value at the D_3_R. NSB and separation are described above. The k_off_ were obtained by applying non-linear regression and fitting to “one phase exponential association” or “two phase exponential association”. As this indirect method underlies model theories and assumptions the resulting k_off_ are approximations that serve mainly as comparison criteria. The effects of Gpp(NH)p, the omitting of sodium-ions in the buffer and of using [^3^H]raclopride as RL were evaluated with the same method (n ≥ 2).

### Measurement of ß-arrestin 2 activation at the D_3_R by aripiprazole and cariprazine

The PathHunter® ß-arrestin eXpress GPCR assay kit was used to measure ß-arrestin recruitment at the D_3_R (protocol of agonists for aripiprazole and cariprazine). Briefly, U2OS cells were incubated at 37 °C for 48 h. Agonist dilutions were added to the respective wells and incubated for 90 min at 37 °C. Afterwards the detection reagent was added and incubated for 60 min at room temperature in the dark. Luminescence was read with the Infinite 1000 Reader (Tecan). Assays ran in duplicates with n = 2 independent experiments. Data was analysed using non-linear regression and fitting to equations “log(agonist) vs. normalized response - variable slope” and “biphasic”. For biphasic fitting “bottom” was constrained to zero, “top” to 100 and nH1/nH2 to 1.

### Molecular modelling approaches at the D_3_R

MD simulations were performed on cariprazine and aripiprazole. The initial structures were constructed from the D_3_R X-Ray structure (Protein database entry code: 3PBL)^32^, mutated residues were transformed to their natural form, eticlopride was removed. The protein was embedded into a 1-palmitoyl-2-oleoyl-sn-glycero-3-phosphocholine (POPC) lipid bilayer, the complexes were solvated in TIP3P waters and neutralized with chloride ions. Parameterization was based on Amber ff14SB force field^33^ and restrained electrostatic potential (RESP)^34^ charges were calculated for the ligands. All preparatory steps were carried out with the default parameters in the BiKi Life Sciences suite^35^. The structures were minimized and equilibrated following the default protocol of the program package (see details in the Supplementary Information). Binding studies against the D_3_R were carried out applying BiKi Life Sciences MD Binding tool implemented in GROMACS-4.6.1^36^ as published recently^37^. This approach is based on adaptive forces attracting the ligand into the predefined binding site, which residues were identified by NanoShaper^38^ and refined by visual inspection (Supplementary Table S1). The additional attractive forces between the ligand and the binding site facilitate the ligand to overcome the barrier of entering the binding pocket; therefore reduce the timescale of the full binding event into a few 10 ns. The bias was switched off, when the ligand reached the 4 Å distance criteria from the backbone heavy atoms of Ser193. During the 20 replica production runs an 0.2 gaining factor was applied in the 30 ns long simulations in isothermal-isobaric (NPT) ensemble at 300 K and 1.013 bar pressure. The binding poses lacking hydrogen bond interaction with the conserved Asp110 were ruled out, the remaining unbiased trajectories were clustered with GROMACS g_cluster tool by the single-linkage method (1.5 Å cut-off) and their stability was tested with scaled MD. The most stable pose was identified as final binding mode and the interaction fingerprints were calculated by IChem^39^ with default settings based on every 10^th^ frame of the corresponding trajectories. The unbinding studies were based on scaled MDs with a scaling factor of 0.4 applying BiKiNetics tool^40^. The final binding mode of the MD binding simulations was the starting pose using structural restraints in canonical (NVT) ensemble at 300 K without the explicit membrane environment. The applied parameters are summarized in Supplementary Table S1. The ligands were considered fully dissociated, when the 6 Å solvation shell around them contained only water molecules.

### Data and statistical analysis

*In vitro* assays were analysed with Prism 6 (GraphPad Software Inc., San Diego, CA) and are given as means ± standard deviation (s.d.). In some instances the number of experiments was increased to characterize binding properties into detail (*e*.*g*. biphasic dissociation of cariprazine). This is marked in the tables. The signal of the indirect kinetic experiments and the ß-arrestin assays were normalized (zero was set as zero) to allow comparison of different curve shapes. Significance of biphasic fitting over one-phasic fitting or “biphasic” over “log(agonist) vs. normalized response” was tested with the globalized data sets using the “extra sum-of-squares F Test” provided by GraphPad. Comparisons were considered significant if p-value was <0.05 (α = 0.05). All *in vitro* assays were performed at room temperature.

### Materials and reagents

[^3^H]Radioligands were purchased from Perkin Elmer (Waltham, USA). Cariprazine was synthesized at RCNS, Hungary. The authors confirm the identity and purity of the given compound. The ß-arrestin kit was purchased from DiscoverX (Birmingham, UK).

## Electronic supplementary material

Supplementary Information

## Data Availability

The datasets generated and analysed during the current study are available from the corresponding author on reasonable request.
