# Supplementary material for: Binding kinetics of cariprazine and aripiprazole at the dopamine D3 receptor
Source: Sci Rep. 2018 Aug 21;8:12509. doi: 10.1038/s41598-018-30794-y (PMC6104066; doi:10.1038/s41598-018-30794-y)
Supplement: Supplementary file 1 — Supplementary Information [file 41598_2018_30794_MOESM1_ESM.pdf]

Supplementary Information

Binding kinetics of cariprazine and aripiprazole at the dopamine D_3 receptor

Annika Frank[1], Dóra J. Kiss[2,3], György M Keserű[2*], Holger Stark[1*]
[1]Institue of Pharmaceutical and Medicinal Chemistry, Heinrich Heine University Düsseldorf, Duesseldorf, Germany
[2]Medicinal Chemistry Research Group, Research Centre for Natural Sciences, Hungarian Academy of Sciences, Budapest, Hungary
[3]ELTE Eötvös Loránd University, Doctoral School of Chemistry, Budapest, Hungary

Equilibration protocol for the MD simulations.

The 5000 steps of steepest descent minimization were followed by three equilibration step in canonical (NVT) ensemble. The temperature was increased from 100 K to 300 K applying the velocity-rescaling temperature coupling available in the GROMACS program package. The backbone restraints were lifted off after the second NVT step. Each stage run for 0.1 ns with 2 fs time steps, except in the first stage a 1 fs time step was applied. The final equilibration step contained 1 ns run in NPT ensemble with 2 fs time steps at 300K and 1.013 bar pressure applying a Parrinello-Rahman barostat.

Table S1: Parameters applied during MD binding and unbinding simulations. Ser193 was set as switch off in the binding simulations

MD protocol	Residues
MD Binding attractive residues	V78 F106 V107 D110 V111 C114 T115 I183 F188 V189 S192 S193 S196 W342 F345 F346 H349 T368 T369 V373
BiKiNetics unrestrained residues	T36 V86 L89 E90 F106 V107 D110 V111 M112 M113 C114 T115 C181 S182 I183 S184 F188 V189 I190 S192 S193 V195 S196 W342 F345 F346 H349 T365 S366 T369 V373

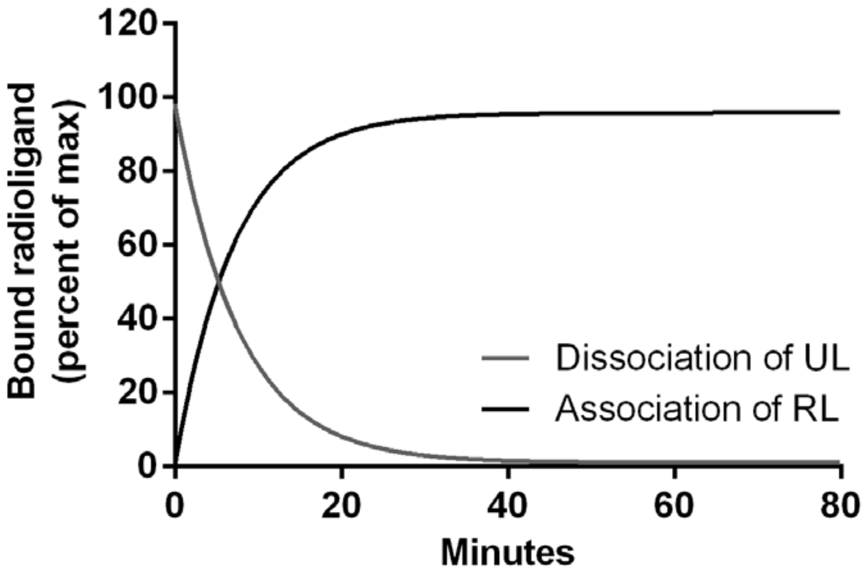

Figure S1: Principle of the dilution method.

Figure Legend

Figure S1: Principle of the dilution method. Receptor preparations are pre-incubated with the UL. Afterwards the dissociation is initiated with an excess of RL. As the RL may only bind, when the UL has dissociated the k_{obs} of the RL equals the k_{off} of the UL.
